# The first step in modern lesion-deficit analysis

**DOI:** 10.1093/brain/awu275

**Published:** 2014-09-29

**Authors:** Parashkev Nachev

**Affiliations:** 1 Institute of Neurology, UCL, London, WC1N 3BG, UK; 2 Institute of Cognitive Neuroscience, UCL, London WC1N 3AR, UK

Sir,

I am grateful to Karnath and his colleague (Karnath and Smith, 2014) for a sophisticated commentary on our recent study (Mah *et al.*, 2014); nonetheless, four aspects of their analysis may cause some readers to misapprehend our conclusions in a way that will tend to perpetuate the errors it was our original aim to correct.

First, the principal reason for changing to multivariate inference is not the complex distributed functional architecture of the brain but the complex distributed *structural* architecture of lesions. Just as mass-univariate inference has not been an obstacle to discovering functional networks with functional MRI, so it would not have been a (major) obstacle to discovering such networks with lesions if lesions had the spatial properties of blood oxygen level-dependent response. Multivariate inference in the context of lesion-mapping is not an extension to the conventional voxel-wise mass-univariate method (i.e. voxel-based lesion–symptom mapping), mainly for those who wish to examine networks as well as single critical areas, but a *necessity* for anyone who uses vascular lesions to do any kind of anatomical inference in the brain. For while the size of the error may well be greater where the pattern of dependence follows a multi-locus, distributed network, substantial error will nonetheless still occur with *single* loci, as we explicitly demonstrate in our paper. We show that the size of such error is sufficient to explain, for example, the surfeit of white matter localisations now crowding the literature.

Second, the large region of interest-based multivariate approach proposed by the authors (Smith *et al.*, 2013) does not solve the problem we have identified but arguably conceals it. We currently do not have robust *functional* criteria for defining large regions of interest—indeed, we need lesion-deficit mapping for this in the first place—and we have shown we cannot easily have robust *anatomical* criteria for defining large regions of interest, based on the architecture of lesions, for the lesion distribution is too complex. Such large scale discretisation will therefore inevitably distort both the putative functional architecture and the lesion architecture, concealing the errors we describe *within* the regions of interest rather than eliminating them. In essence, it transforms the schematized canonical case reproduced in Fig. 1A into the comparably distorting case depicted in Fig. 1B.

**Figure 1:**
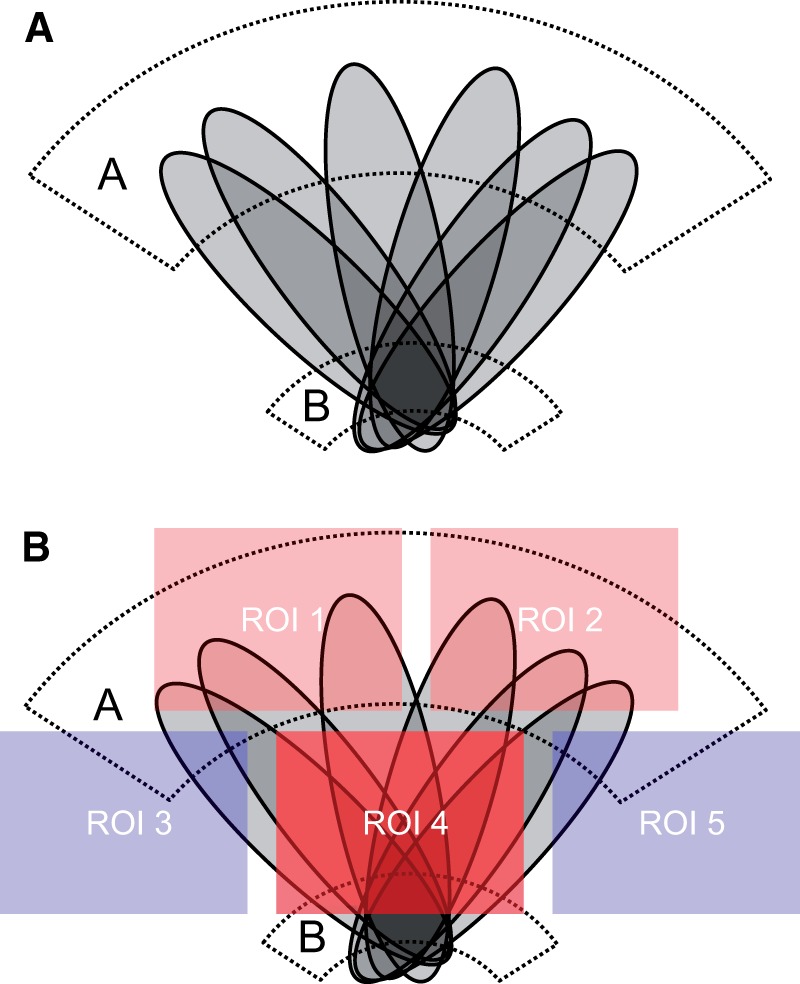
(**A**) Illustration of how stereotyped patterns of brain damage (schematized in grey) across a set of patients can hypothetically mislocalize damage of any part of critical area A (in dotted lines) to the non-critical area B (in dotted lines). This will happen whenever the spatial variability of damage to a non-critical area is *less* for the group or factor of interest than for the critical area. Such stereotypy of damage—a hidden deep structure in the data—may occur where the lesions follow a consistent non-neural architecture, as is the case with vascular lesions. (**B**) Illustration of exactly the same scenario, but now seen through the prism of a large scale discretization into five regions of interest (ROIs), with the colour map indicating the significance of the association with the putative symptom (the more red the stronger). Note that the problem is not only not solved, it is now rendered insoluble by multivariate methods because the biasing effects are concealed within the regions of interest.

Third, although it is self-evident that lesion volume may have an impact on the functional consequences of a lesion, explicitly including it as a regressor in a mass-univariate model will not reduce the error in the inferred critical locus but only amplify it. This is so because lesion volume, in keeping with other summary metrics of lesions, varies with anatomical location, and so will inevitably confound the anatomical inference. For example, as discussed in our paper and elsewhere (Husain and Nachev, 2007), as lesions that reach cortex will generally be larger than subcortical ones such models will unfairly penalize it.

Fourth, the use of continuous behavioural measures, though always to be encouraged, cannot seriously alter a distorting effect rooted in the fundamental architecture of lesions that are naturally careless of their behavioural consequences. Fine behavioural characterization of patients will improve lesion-mapping only if the coarse problems of analysing the underlying anatomy are adequately solved first.

In short, what is needed here is not a rearrangement of the deck chairs, or even a change in their upholstery, but a decisive move to another, very different, ship.

## Funding

The author is funded by the Wellcome Trust and the UCLH Biomedical Research Centre.
